# Obesity in Chronic Obstructive Pulmonary Disease (COPD): Effects on Inflammation, Immune System, Susceptibility to Viral Infections, and Mortality

**DOI:** 10.3390/pathogens15070664

**Published:** 2026-06-23

**Authors:** Laura Vitiello, Stefania Proietti, Dolores Limongi, Carla Prezioso, Caterina Mammi, Massimiliano Caprio, Fabrizio Maggi, Guido Antonelli, Stefano Bonassi, Patrizia Russo

**Affiliations:** 1 Department of Human Sciences and Promotion of the Quality of Life, San Raffaele University, Via di Val Cannuta 247, 00166 Rome, Italy; laura.vitiello@uniroma5.it (L.V.); dolores.limongi@sanraffaele.it (D.L.); carla.prezioso@uniroma5.it (C.P.); massimiliano.caprio@uniroma5.it (M.C.); stefano.bonassi@sanraffaele.it (S.B.); 2 Clinical and Molecular Epidemiology, IRCCS San Raffaele Roma, Via di Val Cannuta 247, 00166 Rome, Italy; 3 AgEA Coordinating Body, 00185 Rome, Italy; stefy7677@gmail.com; 4 Laboratory of Microbiology, IRCCS San Raffaele Roma, Via di Val Cannuta 247, 00166 Rome, Italy; 5 Laboratory of Cardiovascular Endocrinology, IRCCS San Raffaele Roma, Via di Val Cannuta 247, 00166 Rome, Italy; caterina.mammi@sanraffaele.it; 6 National Institute for Infectious Diseases “Lazzaro Spallanzani”-INMI IRCCS, Via Portuense 292, 00149 Rome, Italy; fabrizio.maggi@inmi.it; 7 Department of Molecular Medicine, Sapienza University, 00185 Rome, Italy; guido.antonelli@uniroma1.it; 8 Microbiology and Virology Unit, Sapienza University Hospital Policlinico Umberto I, 00186 Rome, Italy

**Keywords:** COPD, obesity paradox, systemic inflammation, T-cell immunity, Torque Teno virus (TTV), viral biomarkers, oxidative markers, rehabilitation

## Abstract

Chronic obstructive pulmonary disease (COPD) is characterized by systemic inflammation, immune dysregulation, and increased susceptibility to infections. Obesity may influence these processes and has been proposed as a potential contributor to the so-called “obesity paradox”, although its effects on immune competence, viral burden, and survival are not yet fully understood. Seventy patients with severe to very severe COPD (GOLD stage 3–4) were stratified according to BMI (<30 vs. ≥30 kg/m^2^). Clinical and functional parameters were assessed together with biomarkers of oxidative stress, DNA damage, systemic inflammation, and T-cell subsets. A comprehensive viral panel, including Torque Teno virus (TTV), was also analyzed. Five-year survival was evaluated using Kaplan–Meier curves and Cox regression models. Patients with BMI ≥ 30 showed higher lymphocyte counts and increased CD4^+^ and CD8^+^ T-cell levels, accompanied by lower systemic inflammatory indices. No significant differences were observed in oxidative stress or DNA damage markers. In addition, TTV viremia (≥4 log_10_ copies/mL) was more frequently observed among patients with lower BMI. Despite these differences, five-year survival did not significantly differ between the two groups. These findings suggest that BMI alone may have limited value as a predictor of outcomes in patients with advanced COPD. Conversely, immune-inflammatory indices and viral burden, particularly TTV viremia, could provide complementary information for risk assessment and may deserve further investigation as potential tools for personalized patient stratification.

## 1. Introduction

Obesity has become one of the most pressing global public health challenges, currently affecting more than 2 billion adults worldwide and projected to involve over half of the adult population by 2050 [1]. Population aging is expected to further amplify this burden, with nearly one in four individuals with obesity projected to be older than 65 years by mid-century. In high-income countries, the coexistence of increasing obesity prevalence, population aging, and declining fertility rates is likely to place unprecedented pressure on healthcare systems [2].

Although obesity is widely recognized for its cardiometabolic consequences, accumulating evidence indicates that it also profoundly affects immune competence, increasing susceptibility to infectious diseases throughout life [3]. The COVID-19 pandemic provided compelling evidence of this vulnerability, as individuals with obesity experienced disproportionately severe clinical outcomes and higher mortality rates [4]. Large population-based analyses from Finnish cohorts and the UK Biobank, encompassing 925 bacterial, viral, fungal, and parasitic infections, demonstrated that adults with obesity (BMI ≥ 30 kg/m^2^) had a 1.7-fold higher risk of severe infection, rising to nearly threefold among individuals with class III obesity (BMI ≥ 40 kg/m^2^) [3]. Viral infections appeared particularly influenced by excess adiposity, with hazard ratios ranging from 1.3 for herpesvirus infections to 2.3 for acute viral infections. Overall, obesity may account for up to 15% of infection-related deaths worldwide, especially during pandemic periods [5].

The respiratory system is among the organs most profoundly affected by obesity. Excess adiposity induces structural, functional, and immunological alterations that compromise pulmonary physiology and host defense mechanisms [6]. Accumulation of adipose tissue within the thoracic and abdominal compartments mechanically restricts chest wall expansion and diaphragmatic excursion, reducing lung compliance, functional residual capacity (FRC), and expiratory reserve volume (ERV) [7,8,9]. In severe obesity, total lung capacity (TLC) may also decrease, contributing to a restrictive ventilatory pattern [8].

Beyond these mechanical effects, obesity promotes airway remodeling and structural abnormalities. One notable feature is dysanapsis, defined as a mismatch between airway caliber and lung size characterized by relatively smaller airways relative to lung volume [10,11]. This condition is generally associated with preserved or mildly reduced FEV_1_, increased or preserved FVC, and a lower FEV_1_/FVC ratio. Experimental and clinical studies have further demonstrated adipose tissue infiltration within the airways and airway wall thickening, suggesting a direct role of obesity in airway remodeling processes [12,13].

At the microscopic level, obesity induces lipid accumulation within alveolar cells, extracellular matrix deposition, vascular congestion, and alterations in pulmonary immune cell populations [14,15,16]. Increased airway smooth muscle thickness, impaired mucociliary clearance, and chronic low-grade inflammation have also been reported [17,18,19,20]. Adipokines, particularly leptin, appear to contribute substantially to these pathological processes by promoting inflammatory signaling, fibroblast activation, and airway smooth muscle proliferation [21,22].

These structural and inflammatory alterations translate into important functional consequences. Obesity increases airway resistance, respiratory workload, and airway hyper-responsiveness while reducing respiratory endurance and lung volumes [23,24,25,26]. Premature airway closure and ventilation–perfusion mismatch occur more frequently, particularly in dependent lung regions, impairing gas exchange and potentially contributing to the development and progression of chronic respiratory diseases [23,24,25,26]. Respiratory dysfunction is especially pronounced in individuals with central obesity because of the greater mechanical constraint imposed on diaphragmatic movement [27]. Furthermore, obesity-related conditions such as obstructive sleep apnea (OSA) and obesity hypoventilation syndrome (OHS) may further aggravate pulmonary dysfunction through hypoxia-driven inflammatory and vascular pathways [28].

Among chronic respiratory diseases, chronic obstructive pulmonary disease (COPD) represents a particularly relevant condition in which obesity may exert important clinical and biological effects. The relationship between obesity and COPD is complex and partly confounded by smoking, which affects approximately 30–40% of COPD patients and is often associated with lower body weight [29]. Nevertheless, studies conducted in never-smokers have consistently shown that higher BMI is associated with increased COPD risk [30], with central and visceral adiposity exerting stronger effects than generalized obesity [31].

Recent evidence suggests that obesity should be considered not merely a comorbidity but also a potential independent contributor to COPD development and progression [30]. Excess adiposity, particularly abdominal obesity, mechanically restricts lung expansion, increases respiratory workload, and promotes chronic systemic inflammation through adipose tissue dysfunction [32]. Furthermore, a dose–response relationship between obesity severity and COPD risk has been reported among never-smokers, supporting the hypothesis of a direct pathogenic role of obesity in chronic airway disease [30].

In this context, visceral adiposity has emerged as a particularly relevant determinant of respiratory health. A recent study involving 16,167 participants, including 1397 individuals with COPD, demonstrated a significant positive association between the Visceral Adiposity Index (VAI) and COPD prevalence, independent of major confounding factors. This relationship was particularly evident among women and hypertensive individuals, with a threshold effect observed at a VAI value of 2.55 [32]. These findings suggest that markers of visceral adipose dysfunction may improve early risk stratification and facilitate more personalized management strategies in populations at increased risk of COPD.

The obesity–COPD relationship is further complicated by the so-called “obesity paradox,” whereby overweight and moderately obese patients may experience better survival outcomes despite exhibiting worse respiratory symptoms and greater functional impairment [33,34]. The biological mechanisms underlying this paradox remain incompletely understood but likely involve complex interactions among nutritional status, systemic inflammation, body composition, and immune regulation.

Growing evidence suggests that immune dysfunction may represent a central mechanism linking obesity, respiratory disease, and susceptibility to infection. Although the long-standing assumption that the lungs are sterile has been definitively challenged [35], current knowledge of the respiratory virome remains considerably less advanced than that of the respiratory bacteriome. Conventional PCR-based methods have markedly improved the detection of known respiratory viruses; however, most investigations have focused on well-established pathogens such as influenza viruses, respiratory syncytial virus (RSV), and coronaviruses [35]. While these viruses are major causes of acute respiratory infections and exacerbations of chronic lung diseases [35], they likely represent only a small fraction of the diverse viral communities inhabiting the respiratory tract.

The association between obesity and severe viral respiratory infections became particularly evident during the 2009 H1N1 influenza pandemic, when obesity emerged as an independent risk factor for hospitalization, severe disease, and mortality [36,37]. Individuals with severe obesity (BMI ≥ 40 kg/m^2^) were disproportionately represented among influenza-related hospitalizations and deaths, and obesity was consistently associated with poorer clinical outcomes across multiple countries [38]. A World Health Organization (WHO) study involving 20 countries identified obesity, asthma, and pregnancy as major risk factors for severe H1N1 infection [38]. Nevertheless, subsequent investigations yielded conflicting results regarding the relationship between obesity and influenza susceptibility, leaving uncertainty about the precise mechanisms underlying this increased vulnerability [39].

More recently, obesity has been recognized as one of the strongest predictors of severe COVID-19 outcomes. In addition, longitudinal cohort studies have demonstrated that individuals with obesity experience an increased risk of respiratory tract infections during both influenza and non-influenza periods, suggesting a generalized impairment of host defense mechanisms rather than susceptibility to specific viral pathogens alone [40].

Within this framework, increasing attention has been directed toward viruses that persist within the human host without causing overt disease and may therefore serve as indicators of immune competence. Among these, Torque Teno Virus (TTV) has emerged as one of the most promising biomarkers of immune function. TTV is a highly prevalent, non-pathogenic, single-stranded DNA anellovirus that establishes chronic infection in the majority of healthy individuals worldwide [41,42]. Because its replication is largely controlled by the host immune system, circulating TTV levels are believed to reflect the balance between immune surveillance and immune suppression [41,43,44].

The widespread distribution of TTV, together with its stable viral kinetics, low intra-individual variability, and the availability of standardized PCR-based quantification methods, has supported its use as a functional biomarker of immune status in several clinical settings [41,43,44]. In transplant medicine, elevated or increasing TTV DNA levels have been associated with excessive immunosuppression and an increased risk of infectious complications, whereas low viral loads have been linked to acute rejection episodes [43,45]. Similarly, in allogeneic hematopoietic stem cell transplantation, TTV kinetics have been proposed as indicators of immune reconstitution, although interpretation remains influenced by disease severity, conditioning regimens, and lymphocyte recovery dynamics [44].

The potential relevance of TTV extends beyond transplantation. In COPD, TTV may provide valuable insight into the complex interplay among chronic inflammation, immune dysfunction, viral persistence, and clinical outcomes. In the cohort analyzed by Vitiello et al. [46], patients with higher TTV viremia (≥4 log_10_ copies/mL) exhibited significantly lower CD3^+^, CD4^+^, and CD8^+^ T-cell counts, suggesting impaired cellular immune surveillance. Conventional hematological parameters and pulmonary function indices did not differ significantly according to TTV levels, indicating that TTV may capture immunological alterations not reflected by routine clinical markers. Higher TTV loads were also associated with increased frequencies of regulatory T cells (Tregs), and Treg percentages positively correlated with viral load, supporting the hypothesis of an immunosuppressive or immune-tolerant microenvironment. Furthermore, lower TTV viremia was associated with increased expression of the α7 nicotinic acetylcholine receptor (α7nAChR) on CD4^+^ T cells, suggesting a potential link between cholinergic anti-inflammatory pathways and immune control of viral replication.

Collectively, these findings support the concept that TTV is not merely a bystander virus, but rather a surrogate marker of immune competence and immunological balance. Monitoring TTV viral load may therefore help identify COPD patients with impaired immune resilience, contributing to a more comprehensive characterization of host immune status and potentially improving personalized risk stratification strategies in chronic inflammatory lung disease.

The present study aimed to investigate the role of obesity in modulating clinical, immunological, and virological outcomes in elderly patients with advanced COPD. Specifically, the objectives were to: (i) evaluate the impact of body mass index (BMI) on clinical and functional characteristics; (ii) assess oxidative stress and DNA damage as indicators of systemic biological burden; (iii) analyze the relationship between BMI and systemic inflammation, including composite inflammatory indices; (iv) characterize immune profiles, with particular focus on T-cell subsets (CD3^+^, CD4^+^, and CD8^+^); (v) investigate susceptibility to viral infections, including anelloviruses, polyomaviruses, adenoviruses, human herpesviruses, and influenza A/H1N1 virus, with particular emphasis on TTV as a marker of immune competence; and (vi) determine the associations among BMI, immune-inflammatory parameters, viral load, and long-term survival.

By integrating clinical, immunological, and virological data, this study sought to identify novel prognostic markers and contribute to the development of personalized risk stratification strategies in elderly patients with advanced COPD.

## 2. Materials and Methods

### 2.1. Study Design, Participants, and Biological Sample Collection

An observational study was conducted in 70 patients (mean age: 72.6 ± 8.8 years) with severe or very severe chronic obstructive pulmonary disease (COPD; GOLD stage III or IV) admitted to the Pulmonary Rehabilitation (PR) Unit of IRCCS San Raffaele Roma between September 2013 and December 2015 for a comprehensive 3-week pulmonary rehabilitation program.

Peripheral blood samples were collected at admission and centrifuged to obtain plasma and serum, which were stored at −80 °C until analysis.

#### Inclusion and Exclusion Criteria

Eligible participants were adults aged ≥18 years with a confirmed diagnosis of stable COPD. All patients were in a non-acute phase of the disease at the time of enrollment and were admitted for pulmonary rehabilitation.

Exclusion criteria were:

(i) Inability to understand or communicate in Italian;
(ii) Current or previous diagnosis of cancer;
(iii) Mini-Mental State Examination (MMSE) score < 24, indicating significant cognitive impairment; and
(iv) Acute COPD exacerbation at the time of evaluation.

Following plasma separation, the remaining blood fraction was reconstituted with phosphate-buffered saline (PBS) and used for peripheral blood mononuclear cell (PBMC) isolation. PBMCs were separated using 12-mL Leucosep tubes containing Leucosep separation medium (Greiner Bio-One, Merk Life Science S.r.l., Milan, Italy), according to the manufacturer’s instructions. After isolation, PBMCs were washed twice with PBS, resuspended in cryopreservation medium (fetal bovine serum supplemented with 10% dimethyl sulfoxide- all from Sial, Rome, Italy), stored at −80 °C for 2 h, and subsequently transferred to liquid nitrogen for long-term storage. Additional details regarding the study population have been previously reported [47].

A follow-up evaluation was performed by tracing all participants through their municipality of residence. Vital status and causes of death were obtained from municipal registries. The follow-up period extended for 5 years after hospital discharge.

Biological samples were obtained from the biobank of an institutional research project aimed at developing a personalized approach to pulmonary rehabilitation. The study was approved by the Ethics Committee of IRCCS San Raffaele Roma (Protocol No. 15/2013), and all participants provided written informed consent upon admission.

Patient characteristics were assessed through a multidimensional evaluation based on routinely collected clinical, functional, biochemical, and instrumental parameters. Data collected included demographic characteristics (age, sex, marital status, employment status, and educational level), medical history and lifestyle factors (smoking habits, alcohol consumption, family history of COPD and other diseases, and comorbidities), body mass index (BMI), pharmacological treatment, and use of long-term oxygen therapy (LTOT).

Clinical assessment included symptom burden, quality of life, and functional status evaluated using the Borg scale, Medical Research Council (MRC) dyspnea scale, Barthel Index, Activities of Daily Living (ADL/IADL), Maugeri Foundation Respiratory Failure Questionnaire (MRF-26), St. George’s Respiratory Questionnaire (SGRQ), and Short Form-36 (SF-36). Functional exercise capacity was assessed using the six-minute walk test (6MWT), whereas cognitive performance was evaluated using both the Mini-Mental State Examination (MMSE) and the Montreal Cognitive Assessment (MoCA).

Comorbidity burden and clinical complexity were assessed using the Cumulative Illness Rating Scale (CIRS) Severity and Comorbidity Indices, with particular attention to cardiovascular disease and type 2 diabetes mellitus.

Instrumental and physiological evaluations included spirometry (FEV_1_), pulse oximetry, electrocardiography, heart rate, blood pressure, arterial blood gas analysis (pO_2_, pCO_2_, and pH), SpO_2_, and SpCO_2_ measurements. Laboratory assessments included hematological and biochemical parameters, including electrolytes (Na^+^ and K^+^), hemoglobin, glucose, blood urea nitrogen, creatinine, bilirubin, and liver enzymes (ALT, AST, and γGT), as previously described [46].

All patients received daily inhaled corticosteroid therapy (beclomethasone dipropionate 0.4 mg/mL. Chiesi Farmaceutici S.p.A, Parma, Italy) in combination with two bronchodilators (salbutamol and ipratropium bromide all from Chiesi Farmaceutici S.p.A, Parma, Italy) during the 3-week rehabilitation program. Acute exacerbations were managed according to standard clinical practice using systemic corticosteroids, including prednisone, betamethasone, or methylprednisolone.

Smoking status was assessed according to the National Health Interview Survey (NHIS) criteria and verified by urinary cotinine testing at admission. Current smokers were defined as individuals who had smoked at least 100 cigarettes during their lifetime and were actively smoking at the time of evaluation; former smokers were defined as individuals who had smoked at least 100 cigarettes during their lifetime but had quit smoking; and never-smokers were defined as individuals who had never smoked or had smoked fewer than 100 cigarettes during their lifetime. In our cohort, all former smokers had abstained from smoking for at least 10 years. Urine samples were collected at admission, stored at −20 °C, and analyzed for cotinine using a CE-marked Sure Screen Instant Cotinine Testing Kit (ISO 9001 [47] and ISO 13485 [48] certified) (SureScreen Diagnostics, Lucinda House, Sherwood Business Park, Little Oak Drive, NG15 0DR, UK).

### 2.2. Inflammatory Parameters, Oxidative Stress and DNA Damage Assessment, and Flow Cytometry Analysis

#### 2.2.1. Hematological Analysis

Peripheral blood cell counts and percentages were determined as part of routine laboratory testing using an automated hematology analyzer (Beckman Coulter LH500, Beckman Coluter, Cassina de’ Pecchi, Milan, Italy). The lymphocyte-to-monocyte ratio (LMR), neutrophil-to-lymphocyte ratio (NLR), and platelet-to-lymphocyte ratio (PLR) were calculated for each patient using absolute cell counts obtained from complete blood count analyses. Mean values and standard deviations were subsequently calculated.

#### 2.2.2. Biochemical Analysis

Blood samples were centrifuged at 2700× *g* for 10 min. Serum was collected and immediately analyzed. Biochemical assays were performed using a Dade XL^®^ automated analyzer (Siemens Healthcare s.r.l., Milan, Italy) and Dade Behring^®^ reagent kits (Dade Behring Inc., Newark, NJ, USA).

Serum malondialdehyde (MDA) and 8-hydroxy-2′-deoxyguanosine (8-OHdG) concentrations were determined as previously described [46]. Interleukin-6 (IL-6) and C-reactive protein (CRP) levels were measured in serum samples stored at −80 °C for at least one year, according to previously described methods [49].

#### 2.2.3. Alkaline Comet Assay

The complete experimental procedure has been described previously [50]. Briefly, DNA damage in peripheral blood lymphocytes was evaluated following cell lysis, DNA denaturation, agarose gel electrophoresis, and DNA staining using Comet Assay IV software (Instem, London, UK). Tail intensity (TI; % DNA in comet tail) was calculated from 100 comets per individual. A large population study including 8293 subjects reported an overall mean tail intensity of 7.4 ± 8.8%, increasing to 10.5 ± 11.2% among individuals older than 60 years [51].

#### 2.2.4. Flow Cytometry Analysis

Cryopreserved PBMCs were thawed and resuspended in RPMI medium supplemented with 10% fetal bovine serum (FBS), 2 mM L-glutamine, 1% sodium pyruvate, 1% non-essential amino acids, and 1% penicillin/streptomycin (all from SIAL, Rome, Italy) at a concentration of 1 × 10^6^ cells/mL. Cells were seeded in 24-well plates and incubated overnight at 37 °C in a humidified atmosphere containing 5% CO_2_.

To identify lymphocyte populations, cells were stained with the following monoclonal antibodies: anti-CD45 BUV395, anti-CD3 APC-R700, anti-CD4 BUV737, anti-CD8 BV785, anti-CD25 BV421, anti-CD127 BB700, anti-CD183 PE, anti-CD194 BV510, anti-CD196 BV650 (all from BD Biosciences, Milan, Italy), and anti-Nicotinic Acetylcholine Receptor α7 (CHRNA7) Alexa Fluor 488 (Santa Cruz Biotechnology, Dallas, TX, USA).

Following incubation for 20 min at 4 °C, cells were washed, resuspended in PBS, and acquired using an LSR Fortessa X-20 flow cytometer (Becton Dickinson, Milan, Italy). Data were analyzed using FACS Diva software version 8.0.2.

Regulatory T cells (Tregs) and Th17 lymphocytes were identified among CD4^+^ T cells according to the expression of CD25^high CD127^−^ and CD194^+^ CD196^+^ markers, respectively [52,53].

Because lymphocyte immunophenotyping was performed on thawed PBMCs, cell recovery after thawing was insufficient in some samples. Consequently, lymphocyte subset analyses were available for a subset of 50 patients.

### 2.3. Virus Detection and Quantification

Torque Teno Virus (TTV) load was quantified in PBMCs, where the highest viral burden has been reported [54,55]. TTV load was expressed as the number of viral DNA copies per μg of genomic DNA extracted from PBMCs. The lower limit of detection was 10 TTV copies per μg of genomic DNA.

Reference values reported in healthy populations are approximately 2.3 ± 0.7 or 2.8 ± 1.09 log_10_ copies/mL, with no significant sex-related differences [56]. Based on these observations, a TTV load of approximately 4 log_10_ copies/mL has been proposed as a practical threshold indicative of altered immune function [57].

Given the relatively small sample size, patients were stratified into two groups for comparative analyses:

(1) TTV load < 4 log_10_ copies/mL;
(2) TTV load ≥ 4 log_10_ copies/mL.

All other viruses, including influenza A (H1N1), human polyomaviruses (BKPyV and JCPyV), human adenoviruses (Ad11, Ad34, and Ad35), and human herpesviruses (HHV-6, HHV-7, HSV-1, EBV, CMV, and VZV), were detected as previously described [58].

### 2.4. Statistical Analysis

Descriptive statistics are reported as percentages or mean ± standard deviation (SD), as appropriate. Differences between groups were assessed using Student’s *t*-test or the Mann–Whitney U test.

Survival time was defined as the interval (days) between baseline assessment and death. Participants who were alive at the end of follow-up were censored.

Cox proportional hazards regression models were used to evaluate associations between clinical variables and survival outcomes. The proportional hazards assumption was assessed using Schoenfeld residuals (cox.zph), and no major violations were observed in the final models. Hazard ratios (HRs) and corresponding 95% confidence intervals (CIs) were reported.

The study was exploratory in nature, aiming to explore a wide range of potential associations and hypotheses, rather than testing specific pre-defined hypotheses. Therefore, we decided not to apply Bonferroni correction, which is more suitable for confirmatory studies with a limited number of a priori hypotheses, while in exploratory research an overly stringent correction like Bonferroni might steal the identification of potentially important findings.

Normality was assessed using the Kolmogorov–Smirnov test. For biomarkers with a well-established biological tendency toward symmetric distributions (e.g., blood cell counts), biological plausibility was also considered when interpreting normality assumptions. Variables that did not meet normality criteria were analyzed using non-parametric methods.

Statistical significance was established at α = 0.05. All analyses were performed using R software (version 4.5.2) with the survival and survminer packages.

## 3. Results

### 3.1. The COPD Cohort Analysis

A total of 70 participants were included in the baseline analysis (Table 1). Participants were stratified according to BMI (<30 vs. ≥30 kg/m^2^) to investigate systemic inflammation through inflammatory indices (NLR, PLR, MLR, SII, SIRI, and AISI), immunological profiles (CD3^+^ T lymphocytes), DNA damage (% tail intensity assessed by comet assay), oxidative stress markers (8-OHdG and malondialdehyde), susceptibility to viral infections, response to pulmonary rehabilitation, and 5-year survival.

Of the study cohort, 50 patients (71.4%) had a BMI < 30 kg/m^2^, whereas 20 patients (28.6%) were classified as obese (BMI ≥ 30 kg/m^2^) according to World Health Organization criteria.

The mean age of the cohort was 70.5 ± 8.3 years. Patients with BMI < 30 kg/m^2^ were significantly older than those with BMI ≥ 30 kg/m^2^ (71.8 ± 8.6 vs. 67.2 ± 6.8 years, respectively; *p* = 0.033). All patients had severe or very severe COPD (GOLD stage III or IV).

Patients with BMI ≥ 30 kg/m^2^ exhibited numerically higher FEV_1_/FVC values than patients with BMI < 30 kg/m^2^ (55.6 ± 24.5 vs. 43.4 ± 20.7; *p* = 0.090). Similarly, the obese group showed longer six-minute walk test (6MWT) distances (120.5 ± 84.7 vs. 102.2 ± 85.9 m; *p* = 0.422), although the difference did not reach statistical significance.

Cognitive performance assessed by MMSE and functional autonomy evaluated by ADL and IADL scores were comparable between groups (Table 2). In contrast, patients with BMI ≥ 30 kg/m^2^ showed significantly higher BDI-II scores than patients with BMI < 30 kg/m^2^ (16.0 ± 10.2 vs. 9.7 ± 4.9, respectively; *p* = 0.049), indicating a greater burden of depressive symptoms.

Participants were also stratified according to smoking status (current, former, and never smokers). No significant differences in smoking distribution were observed between BMI groups (*p* = 0.782).

### 3.2. Oxidative Stress and DNA Damage Markers

No significant differences were observed between BMI groups for oxidative stress or DNA damage markers (Table 3).

Specifically, serum malondialdehyde (MDA) concentrations were 40.21 ± 12.47 μM in patients with BMI ≥ 30 kg/m^2^ and 41.51 ± 13.04 μM in patients with BMI < 30 kg/m^2^ (*p* = 0.767). Likewise, serum 8-hydroxy-2′-deoxyguanosine (8-OHdG) levels did not differ significantly between groups (23.93 ± 9.91 vs. 25.40 ± 12.57 pg/mL; *p* = 0.721).

Similarly, IL-6 concentrations were comparable in obese and non-obese patients (124.51 ± 155.50 vs. 108.78 ± 136.45 pg/mL; *p* = 0.778). DNA damage assessed by comet assay (% tail intensity) was also similar between groups (20.11 ± 8.24% vs. 19.62 ± 7.52%; *p* = 0.826).

### 3.3. Hematological, Immunological and Inflammatory Markers

The hematological, immunological, and inflammatory profiles of the study population are summarized in Table 4, Table 5 and Table 6.

No significant differences were observed in routine hematological parameters, including red blood cell count (RBC; *p* = 0.359), white blood cell count (WBC; *p* = 0.506), or platelet count (PLT; *p* = 0.227).

In contrast, absolute lymphocyte counts were significantly higher in patients with BMI ≥ 30 kg/m^2^ than in those with BMI < 30 kg/m^2^ (2.9 ± 2.7 vs. 1.5 ± 0.8 × 10^3^/mL; *p* = 0.001).

Inflammatory indices were significantly influenced by BMI status (Table 5). IL-6, CRP, and LMR did not differ significantly between groups (*p* = 0.778, *p* = 0.108, and *p* = 0.382, respectively).

Conversely, NLR and PLR were significantly lower in obese patients, showing reductions of 50.6% (*p* = 0.001) and 49.8% (*p* = 0.002), respectively. MLR also showed a lower value in the obese group, although the difference did not reach statistical significance (−38.1%; *p* = 0.066).

Similarly, the Systemic Immune-Inflammation Index (SII) was reduced by 41% (*p* = 0.003), the Systemic Inflammation Response Index (SIRI) by 57.1% (*p* = 0.046), and the Aggregate Index of Systemic Inflammation (AISI) by 45.1% (*p* = 0.051) in patients with BMI ≥ 30 kg/m^2^.

No significant differences were observed for the Albumin-Bilirubin (ALBI) score (*p* = 0.392) or the Platelet-Albumin-Bilirubin (PALBI) score (*p* = 0.232).

Analysis of T-cell subsets revealed significantly higher numbers of circulating CD3^+^ T lymphocytes in patients with BMI ≥ 30 kg/m^2^ compared with those with BMI < 30 kg/m^2^ (*p* = 0.025). Significant increases were also observed for both CD4^+^ T cells (*p* = 0.030) and CD8^+^ T cells (*p* = 0.032) (Table 6).

No significant differences were detected in the CD4/CD8 ratio (*p* = 0.216), the percentage of regulatory T cells (Tregs; *p* = 0.725), Th17 cells (*p* = 0.268), or the Treg/Th17 ratio (*p* = 0.450).

### 3.4. Virological Status Analysis

Virological findings are summarized in Table 7. A broad panel of viruses was investigated, including influenza A (H1N1), human polyomaviruses (BKPyV and JCPyV), human adenoviruses (Ad11, Ad34, and Ad35), human herpesviruses (HHV-6, HHV-7, HSV-1, EBV, CMV, and VZV), and members of the Anelloviridae family, including Torque Teno Virus (TTV).

To exclude evidence of ongoing hepatotropic viral infection, serum alanine aminotransferase (ALT), aspartate aminotransferase (AST), and gamma-glutamyl transferase (γGT) levels were evaluated (Table 4). All values were within normal laboratory ranges and did not differ significantly between BMI groups.

Among all viruses tested, only TTV viremia > 4 log_10_ copies/mL was significantly associated with BMI category (OR = 0.32, 95% CI: 0.11–0.93; *p* = 0.040), with a higher prevalence observed among patients with BMI < 30 kg/m^2^.

No significant associations were observed between BMI status and the presence of polyomaviruses, adenoviruses, herpesviruses, or influenza A/H1N1.

### 3.5. Survival

**Figure 1:**
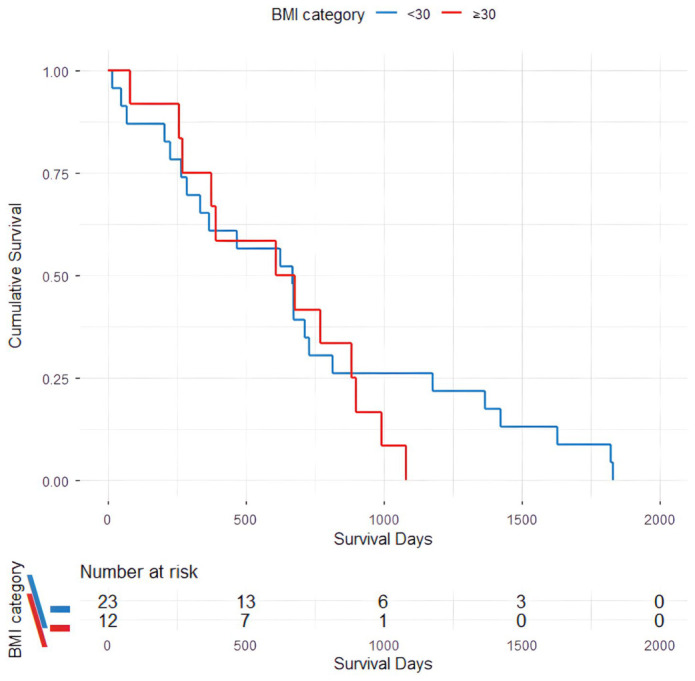
Kaplan–Meier survival curve according to BMI category.

Kaplan–Meier survival analysis was performed to compare overall survival between patients with BMI < 30 kg/m^2^ and those with BMI ≥ 30 kg/m^2^ over approximately 2000 days of follow-up (Figure 1).

No statistically significant difference in survival was observed between the two BMI categories (log-rank *p* = 0.48). This finding was consistent with the substantial overlap of the corresponding 95% confidence intervals throughout the observation period.

Mean survival time was 714.2 ± 564.8 days in patients with BMI < 30 kg/m^2^ and 607.2 ± 493.5 days in patients with BMI ≥ 30 kg/m^2^.

In the multivariable Cox model including BMI category and age, neither variable showed a significant association with survival. BMI ≥ 30 had an HR of 1.08 (95% CI 0.49–2.39; *p* = 0.84), and age had an HR of 0.97 (95% CI 0.92–1.02; *p* = 0.18). The model’s discrimination was limited (concordance = 0.58), and all global tests were non-significant (*p* = 0.30). Because all 39 subjects experienced the event with no censoring, the model is statistically unstable and cannot provide reliable adjusted estimates. These results indicate that neither BMI category nor age can be interpreted as independent prognostic factors in this dataset.

Overall, survival distributions were comparable between BMI groups, and obesity was not significantly associated with long-term mortality in this cohort of patients with advanced COPD undergoing pulmonary rehabilitation.

Kaplan–Meier estimates of cumulative survival in patients with COPD undergoing pulmonary rehabilitation, stratified according to BMI (<30 kg/m^2^ vs. ≥30 kg/m^2^). Survival time was calculated from study entry to death or censoring. Both groups exhibited a progressive decline in survival probability during follow-up, with substantial overlap of the survival curves. Tick marks indicate censored observations. The number of patients at risk at predefined time points is reported below the *x*-axis.

## 4. Discussion

This study provides further insight into the complex interplay among BMI, systemic inflammation, immune status, oxidative stress, viral burden, and survival in patients with advanced COPD. In our cohort, characterized by severe airflow limitation, a high burden of multimorbidity, and advanced age, BMI alone did not emerge as a dominant determinant of prognosis. Survival analysis showed largely overlapping Kaplan–Meier curves between BMI categories (<30 vs. ≥30 kg/m^2^), with no statistically significant difference (*p* = 0.48), indicating comparable long-term survival in patients with lower and higher BMI. Although individuals with BMI ≥ 30 kg/m^2^ initially exhibited slightly higher survival probabilities, this apparent advantage was not sustained over time, and the survival curves ultimately converged. These findings suggest that BMI alone does not independently determine prognosis in this population. These results are partially consistent with the concept of the “obesity paradox” in COPD, according to which overweight or moderately obese individuals often exhibit more favorable biological and clinical profiles than lean patients [34,59,60,61,62,63,64].

**Figure 2:**
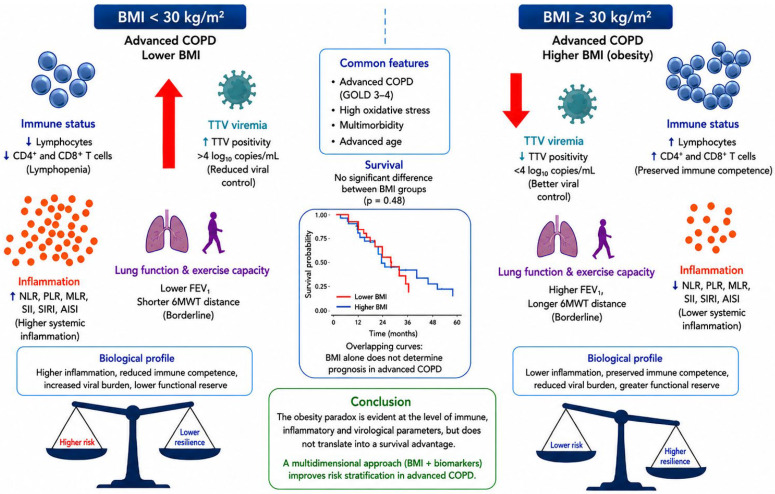
Proposed biological mechanisms linking BMI, immune function, inflammation, functional status, and survival in advanced COPD.

In our cohort, patients with BMI ≥ 30 kg/m^2^ demonstrated a more favorable immunological, biochemical, and virological profile, including higher lymphocyte and T-cell counts, lower systemic inflammatory indices (NLR, PLR, MLR, SII, SIRI, and AISI), and reduced TTV viremia, suggesting better immune competence and viral control (Figure 2). In this respect, our findings are consistent with the obesity paradox at the biological level, supporting the notion that higher BMI in advanced COPD may be associated with preserved immune function and a comparatively attenuated inflammatory state. However, these differences were not reflected in survival outcomes, as no significant differences were observed between BMI groups. Therefore, in this elderly population with advanced COPD, the obesity paradox appears to be context-dependent, manifesting primarily in immunological and virological parameters without translating into a measurable survival advantage.

Chronic obstructive pulmonary disease (COPD) is characterized by persistent systemic inflammation and immune dysregulation. In the present cohort, patients with BMI < 30 kg/m^2^ exhibited a less favorable biological profile, including higher levels of inflammatory markers (NLR, SII, PLR, MLR, and related systemic inflammation indices), lower lymphocyte counts, and increased Torque Teno Virus (TTV) viremia, suggesting impaired immune competence and a greater viral burden. These patients also showed poorer pulmonary function and reduced exercise capacity, reflected by shorter six-minute walk distances (6MWD), consistent with greater tissue wasting, reduced physiological reserve, and increased disease severity.

Conversely, patients with BMI ≥ 30 kg/m^2^ demonstrated a comparatively preserved immunological profile, characterized by higher lymphocyte counts, lower inflammatory marker levels, and reduced TTV viremia. Obese patients also exhibited better lung function and exercise performance, suggesting greater metabolic and functional reserves despite advanced COPD. These findings are consistent with the obesity paradox hypothesis, whereby increased body mass may provide protective physiological reserves in chronic diseases.

Despite these biological and functional differences, Kaplan–Meier survival analysis revealed no statistically significant differences in long-term survival between BMI groups, with substantial overlap of the survival curves throughout follow-up. This observation suggests that BMI alone is insufficient to explain prognosis in advanced COPD. Rather, mortality is likely influenced by the complex interaction among age, disease severity, systemic inflammation, immune competence, comorbidity burden, nutritional status, and functional capacity. Overall, these findings indicate that BMI modulates the inflammatory and immunological phenotype of advanced COPD without translating into measurable differences in long-term survival. A multidimensional approach integrating anthropometric, functional, inflammatory, virological, and clinical variables may therefore provide a more accurate assessment of prognosis and risk stratification than BMI alone. Figure has been improved using ChatGPT. (ChatGPT plus (GPT-5.5)).

In our cohort, patients with BMI ≥ 30 kg/m^2^ tended to exhibit slightly higher FEV_1_ values and longer six-minute walk test (6MWT) distances than non-obese individuals, although these differences did not reach statistical significance. These observations are consistent with previous reports suggesting greater functional reserve in overweight or obese patients with COPD [59]. Data from the large international SUMMIT trial further support a non-linear relationship between BMI and mortality in COPD, with the highest mortality observed at the extremes of body weight—underweight (<20 kg/m^2^) and severe obesity (≥40 kg/m^2^)—and lower mortality among moderately overweight individuals [64].

Mechanistically, low BMI in advanced COPD is often associated with muscle wasting, systemic catabolism, and cachexia, all of which contribute substantially to morbidity and mortality [65,66,67]. Conversely, higher BMI may reflect greater energy reserves capable of buffering the metabolic demands imposed by chronic respiratory disease. According to the muscle mass hypothesis, metabolically healthy overweight or obese patients with preserved lean mass may better tolerate acute illness and systemic inflammatory stress [68,69,70]. Importantly, BMI does not distinguish between metabolically healthy adiposity and sarcopenic obesity [71], highlighting the need for more refined body composition phenotyping.

With regard to oxidative stress, no significant differences were observed between BMI groups, in contrast with studies in the general population linking obesity to increased oxidative burden [37]. Similarly, after stratification according to smoking status (current, former, and never smokers), no significant differences were detected among smoking categories, therefore it was not retained in the final multivariable analyses.

Markers of oxidative stress and inflammation—including malondialdehyde, 8-hydroxy-2′-deoxyguanosine, interleukin-6, and DNA damage—were markedly elevated across the entire cohort compared with healthy populations, suggesting that the high baseline oxidative and inflammatory burden characteristic of advanced COPD may mask any additional contribution of adiposity or smoking status. For example, urinary 8-OHdG levels are typically approximately 8.1 ng/mL in healthy individuals and 12.2 ng/mL in COPD patients [39], whereas mean values in our cohort exceeded 23 ng/mL. Similarly, IL-6 concentrations in healthy populations are generally below 6–7 pg/mL [40], whereas our cohort exhibited mean levels of 77.7 ± 126.7 pg/mL.

These findings suggest that, in advanced COPD, systemic oxidative stress is predominantly disease-driven rather than BMI- or smoking-dependent. Consistent with this interpretation, bilirubin—an endogenous antioxidant capable of scavenging peroxyl radicals and inhibiting NADPH oxidase activity [72]—did not differ between BMI groups despite previous reports linking higher bilirubin levels with improved lung function and reduced mortality in COPD [73]. Although long-term effects of smoking on oxidative stress, inflammation, and immune function have been widely reported [74], no significant differences were observed among smoking categories in the present cohort.

An additional key finding was the significant reduction in several systemic inflammatory indices—including NLR, PLR, MLR, SII, SIRI, and AISI—in patients with BMI ≥ 30 kg/m^2^. These indices are increasingly used as markers of systemic inflammation and have demonstrated prognostic value across multiple diseases. SII, originally developed in oncology, reflects neutrophil–platelet–lymphocyte interactions and predicts outcomes in hepatocellular carcinoma and lupus nephritis [75,76], whereas AISI has shown prognostic relevance in stroke, hypertension, idiopathic pulmonary fibrosis, and COPD [77]. Similarly, SIRI has been associated with COPD incidence and progression [78]. In our cohort, SIRI values were generally elevated (mean 4.20 ± 3.92), reflecting the systemic inflammatory state characteristic of advanced COPD. Nevertheless, patients with BMI ≥ 30 kg/m^2^ exhibited significantly lower values than those with BMI < 30 kg/m^2^ (*p* = 0.046). Notably, PLR was markedly reduced (−49.8%) in obese patients (*p* = 0.002). This consistent reduction across inflammatory indices may indicate a comparatively attenuated inflammatory phenotype, potentially reflecting preserved nutritional status or differences in immune regulation.

Regarding immune function, we observed substantial differences in T-lymphocyte counts between BMI groups. Patients with BMI < 30 kg/m^2^ exhibited markedly lower lymphocyte counts, whereas patients with BMI ≥ 30 kg/m^2^ showed normal to elevated lymphocyte levels. Specifically, CD4^+^ and CD8^+^ T-cell counts were significantly higher in obese patients, while the CD4/CD8 ratio and regulatory T-cell populations remained unchanged. These findings suggest that obesity in this clinical context is associated with preservation of T-cell populations without evidence of overt immune dysregulation.

The advanced age of the cohort is particularly relevant, as aging is associated with immunosenescence, characterized by thymic involution, contraction of the naïve T-cell pool, and expansion of memory and cytotoxic subsets [79]. Adipose tissue may partially counterbalance these processes through immunometabolic signaling, as it is increasingly recognized as an active immunological organ that secretes adipokines and cytokines capable of modulating immune cell development and survival [80,81]. Mediators such as leptin, IL-6, and TNF-α influence T-cell proliferation, survival, and differentiation, potentially supporting lymphocyte homeostasis even in older individuals. Furthermore, moderate increases in BMI among elderly populations may reflect better nutritional status and greater protein availability for lymphopoiesis [82], whereas lower BMI may indicate sarcopenia, frailty, or subclinical malnutrition, conditions strongly associated with lymphopenia and impaired immune responses [83].

Virological analysis further revealed that, among all viruses investigated, only TTV viremia was significantly associated with BMI. TTV positivity (>4 log_10_ copies/mL) was significantly more frequent among patients with BMI < 30 kg/m^2^, suggesting reduced immune control in leaner individuals. TTV is increasingly recognized as a surrogate biomarker of immune competence, with higher viral loads reflecting impaired immune surveillance [84]. Previous work from our group demonstrated that elevated TTV viremia predicts poor survival in COPD patients [55].

Taken together, these findings indicate that BMI alone is an insufficient predictor of outcomes in advanced COPD. Rather, a multidimensional approach integrating immune-inflammatory indices and viral biomarkers may provide a more accurate assessment of biological vulnerability. Lower BMI identified a subgroup characterized by higher systemic inflammation, reduced lymphocyte counts, and increased TTV positivity, suggesting impaired immune resilience.

Integrating BMI with biomarkers such as PLR and TTV load may therefore improve risk stratification and guide personalized rehabilitation strategies. PLR, which reflects both inflammatory and coagulation pathways, is particularly attractive because of its low cost and availability from routine blood testing. Platelet-derived mediators, including platelet-activating factor, may exacerbate airway inflammation and promote thrombosis, impairing pulmonary circulation and contributing to post-rehabilitation exacerbations. A recent study identified PLR, eosinophil percentage, and NLR as among the most reliable predictors of acute exacerbation risk following pulmonary rehabilitation [85].

TTV measurement is also feasible in clinical practice, as commercially available assays enable relatively simple and cost-effective testing. Together, these biomarkers may offer a favorable cost–benefit profile for identifying high-risk COPD patients and tailoring interventions aimed at improving nutritional status, immune competence, and long-term outcomes.

Overall, these findings reinforce the concept of COPD as a systemic disease in which metabolic status, immune regulation, and viral surveillance interact dynamically to shape clinical outcomes.

### Strengths and Limitations

This study has several strengths. First, it provides a multidimensional characterization of patients with advanced COPD by integrating clinical, functional, inflammatory, oxidative, immunological, and virological parameters within the same cohort. This comprehensive approach enabled the exploration of the complex interactions among BMI, systemic inflammation, immune competence, and viral burden beyond the traditional evaluation of anthropometric measures alone.

Second, the study incorporated detailed immune profiling, systemic inflammatory indices (NLR, PLR, MLR, SII, SIRI, and AISI), and TTV viremia, an emerging biomarker of immune competence. The integration of these parameters enabled the identification of immune-inflammatory and virological features associated with biological vulnerability beyond BMI.

However, several limitations should be acknowledged. First, this was a single-center observational cohort study conducted in a relatively small sample of 70 elderly patients with advanced COPD, of whom only 20 had a BMI ≥ 30 kg/m^2^. This limited sample size, particularly within the obesity subgroup, may have reduced the statistical power to detect differences in clinical outcomes and may limit the generalizability of the findings.

Second, BMI was used as the primary anthropometric measure and does not distinguish between fat mass and lean body mass, thereby limiting the interpretation of body composition effects. In particular, the absence of a comprehensive body composition assessment may have prevented a more accurate characterization of the relationships among adiposity, muscle mass, and clinical outcomes. However, it should be noted that this study was conducted in a real-world pulmonary rehabilitation setting, and detailed body composition analyses are not routinely performed in clinical practice within the regional rehabilitation pathways currently adopted according to Lazio Regional Health Service guidelines [86].

Furthermore, because lymphocyte immunophenotyping was performed on thawed samples and, in some cases, the number of viable cells recovered after thawing was insufficient for analysis, lymphocyte subset characterization could only be performed in a subset of 50 patients. This may have reduced the statistical power of the immunological analyses and increased the risk of a type II error.

In addition, adjustment for potential confounding factors was necessarily limited by the sample size, and residual confounding cannot be excluded. Finally, the cohort consisted predominantly of elderly patients with advanced COPD (GOLD stages 3–4), which may limit the applicability of these findings to younger populations or to patients with milder stages of the disease.

Despite these limitations, this study provides preliminary insights into the complex relationships among body mass, immune-inflammatory status, and viral burden in elderly patients with advanced COPD. Our findings suggest that immune-inflammatory and virological biomarkers may be associated with biological vulnerability more consistently than BMI alone. While the observed immunological, biochemical, and virological profiles were broadly consistent with the concept of an “obesity paradox,” these differences were not accompanied by significant survival advantages across BMI categories in this cohort. Given the exploratory nature of the study and its limited sample size, these observations should be interpreted with caution. Nevertheless, they highlight the potential value of integrating virological and immune-inflammatory markers with traditional clinical assessments to improve patient characterization and risk stratification in advanced COPD.

## 5. Conclusions and Future Directions

In this cohort study of 70 elderly patients with advanced COPD, including 20 individuals with a BMI ≥ 30 kg/m^2^, viral burden—particularly elevated Torque Teno Virus (TTV) load—and immune-inflammatory markers such as the platelet-to-lymphocyte ratio (PLR) appeared to be associated with biological vulnerability more consistently than BMI alone. Although patients with higher BMI tended to exhibit a more favorable immune-inflammatory and virological profile, these differences were not accompanied by significant survival advantages during follow-up.

Markers of oxidative stress and DNA damage were elevated across the cohort, suggesting that the systemic burden associated with advanced COPD may partially outweigh potential BMI-related influences on clinical outcomes. In this context, our findings are consistent with the hypothesis that the so-called “obesity paradox” in COPD may be context-dependent. While higher BMI was associated with more favorable immunological and virological characteristics, this pattern did not translate into measurable survival benefits in the present population.

Taken together, these observations suggest that immune competence and viral burden may contribute to the heterogeneity of clinical outcomes in advanced COPD and may provide complementary information beyond traditional anthropometric measures. From a rehabilitation perspective, these findings further suggest that BMI alone may not fully capture the biological complexity and vulnerability of patients undergoing pulmonary rehabilitation. The integration of immune-inflammatory and virological parameters into clinical assessment could help improve patient phenotyping and identify subgroups with differing degrees of physiological reserve and risk.

However, these findings should be interpreted with caution, given the observational cohort design, the relatively small sample size, and the limited number of patients with obesity. These factors may have reduced the statistical power to detect differences in survival outcomes and may limit the generalizability of the results. Moreover, the study was not specifically designed to evaluate the impact of obesity on mortality.

Further prospective studies involving larger and more balanced cohorts are warranted to clarify the complex interplay among body composition, immune competence, systemic inflammation, oxidative stress, and viral burden in COPD. The integration of viral monitoring, immune profiling, and more detailed assessments of body composition may help refine patient characterization and support the development of more personalized clinical and rehabilitation strategies for patients with advanced COPD.

## Figures and Tables

**Table 1:** Demographical and clinical features of COPD patients, stratified by BMI.

Variable	All Patients	BMI < 30 50 (71.4%)	BMI ≥ 30 20 (28.6%)	*p* Value
Age at admission	70.5 ± 8.3	71.8 ± 8.6	67.2 ± 6.8	0.033
Sex (F/M)	34/36	23/27	11/9	0.677
BMI	27.3 ± 7.9	23.3 ± 3.5	37.1 ± 7.2	<0.001
Education (years)				
5	22 (31.4%)	16 (32%)	6 (20%)	
8	23 (32.8%)	15 (30%)	8 (40%)	0.782
10–18	25 (35.7%)	19 (38%)	6 (20%)	
Smoking				
Current	29 (41.4%)	22 (44%)	7 (35%)	
Former	32 (45.7%)	22 (44%)	10 (50%)	0.782
Never	9 (12.9%)	6 (12%)	3 (15%)	
Clinical features				
6MWT (m)	107.43 ± 85.40	102.20 ± 85.9	120.50 ± 84.7	0.422
Barthel Index (normal value 100)	73.2 ± 19.0	72.9 ± 18.3	73.2 ± 19.0	0.849
Borg Scale	7.81 ± 0.9	7.86 ± 0.9	7.70 ± 0.9	0.508
MRC	4.0 ± 0.0	4.0 ± 0.0	4.0 ± 0.0	--
MRF-26	71.4 ± 14.4	70.9 ± 15.2	72.8 ± 12.2	0.635
StGeorge Score (%)	47.3 ± 15.9	45.9 ± 14.9	51.1 ± 18.0	0.246
FEV1/FVC%	47.2 ± 22.4	43.4 ± 20.7	55.6 ± 24.5	0.09
CIRS severity	1.5 ± 0.2	1.5 ± 0.2	1.5 ± 0.2	0.663
CIRS comorbidity	2.5 ± 1.3	2.4 ± 1.3	2.7 ± 1.5	0.414
Survival (days)	677.5 ± 493.5	714.22 ± 564.8	607.2 ± 493.5	0.550

Variables were measured at patients’ admission. Abbreviations: 6MWT, six minute walking test; MRC, modified Medical Research Council (Dyspnea Scale); MRF-26, Maugeri Respiratory Failure 26 questions; FEV1/FVC%, Forced Expiratory Volume in the first second/Forced Vital Capacity (ratio expressed as percentage); CIRS, Cumulative Illness Rating Scale.

**Table 2:** Cognitive, psychological and QoL (Quality-of-Life) values, measured at patients’ admission.

Variable	All Patients	BMI < 30 50 (71.4%)	BMI ≥ 30 20 (28.6%)	*p* Value
ADL	5.13 ± 1.5	5.2 ± 1.5	5.0 ± 1.6	0.671
IADL	12.0 ± 3.8	11.9 ± 4.2	12.1 ± 3.3	0.925
MMSE	27.1 ± 2.9	27.3 ± 2.9	26.7 ± 3.1	0.479
BDI-II	11.2 ± 6.9	9.7 ± 4.9	16.0 ± 10.2	0.049
CES-D	11.6 ± 10.6	11.2 ± 6.9	10.9 ± 10.2	0.453
ZUNG	29.4 ± 9.0	28.4 ± 8.1	32.1 ± 10.9	0.161
SF-36 General Health (QoL)	72.3 ± 12.5	73.5 ± 12.6	68.9 ± 11.9	0.241
SF-36 Mental Health (QoL)	62.9 ± 9.9	63.6 ± 8.1	60.8 ± 14.1	0.379

Abbreviations: ADL, Activities of Daily Living; IADL, Instrumental Activities of Daily Living; MMSE, Mini-Mental State Examination; BDI-II, Beck Depression Inventory-II; CES-D, Center for Epidemiologic Studies Depression Scale; ZUNG, Zung Self-Rating Depression Scale; SF-36 General Health (QoL), 36-Item Short Form Health Survey—General Health domain; SF-36 Mental Health (QoL), 36-Item Short Form Health Survey—Mental Health domain.

**Table 3:** DNA damage and oxidative stress markers analysis.

Variable	All Patients	BMI < 30 50 (71.4%)	BMI ≥ 30 20 (28.6%)	*p* Value
Comet Assay (Tail intensity)	19.7 ± 7.6	19.6 ± 7.5	20.1 ± 8.2	0.826
MDA (Malondialdehyde)	41.1 ± 12.7	41.5 ± 13.0	40.2 ± 12.5	0.767
8-hydroxy-2′ -deoxyguanosine (OH8dG)	24.9 ± 11.7	25.4 ± 12.6	23.9 ± 9.9	0.721

**Table 4:** Blood analysis parameters.

Variable	All Patients	BMI < 30 50 (71.4%)	BMI ≥ 30 20 (28.6%)	*p* Value
RBC (×10^6^/mL)	4.4 ± 0.7	4.4 ± 0.7	4.5 ± 0.7	0.359
Hgb (g/dL)	12.8 ± 1.8	12.6 ± 1.8	13.4 ± 1.6	0.011
PLT (×10^3^/mL)	259.9 ± 96.5	268.9 ± 105.0	237.7 ± 68.8	0.227
WBC (×10^3^/mL)	10.7 ± 3.9	10.5 ± 3.9	11.2 ± 4.4	0.506
Neutrophils (×10^3^/mL)	8.0 ± 3.6	8.2 ± 3.6	7.7 ± 3.5	0.636
Eosinophils (×10^3^/mL)	0.1 ± 0.1	0.1 ± 0.1	0.1 ± 0.1	0.122
Basophils (×10^3^/mL)	0.02 ± 0.02	0.02 ± 0.02	0.02 ± 0.02	0.433
Lymphocytes (×10^3^/mL)	1.9 ± 1.7	1.5 ± 0.8	2.9 ± 2.7	0.001
ESR mm/h	28.5 ± 28.8	31.3 ± 30.3	21.6 ± 24.6	0.894
Blood glucose (mg/dL)	110.3 ± 54.6	109.0 ± 53.1	114.0 ± 61.5	0.797
Azotemia (mg/dL)	54.8 ± 25.8	56.4 ± 28.9	51.2 ± 17.8	0.478
Creatinine (mg/dL)	0.9 ± 0.3	0.9 ± 0.3	0.9 ± 0.3	0.998
ALT (UI/L)	17.6 ± 9.7	16.4 ± 9.2	20.1 ± 10.7	0.269
AST (UI/L)	22.0 ± 12.5	20.3 ± 12.9	25.5 ± 11.3	0.229
γGT (UI/L)	41.6 ± 42.7	38.9 ± 47.8	47.0 ± 30.9	0.586
Albumin (g/dL)	3.9 ± 0.4	3.8 ± 0.3	3.9 ± 0.5	0.324

Abbreviations: RBC, Red Blood Cells; PLT, platelets; WBC, white blood cells; ESR, erythro sedimentation rate; ALT, alanine aminotransferase; AST, aspartate aminotransferase; γGT, gamma-glutamyl transferase.

**Table 5:** Inflammation markers.

Variable	All Patients	BMI < 30 50 (71.4%)	BMI ≥ 30 20 (28.6%)	*p* Value
IL-6 (pg/mL)	113.1 ± 139.6	108.8 ± 136.4	124.5 ± 155.5	0.778
CRP (mg/dL)	1.4 ± 2.6	1.9 ± 3.2	0.5 ± 0.6	0.108
LMR	2.9 ± 2.2	2.7 ± 2.4	3.2 ± 1.8	0.382
MLR	0.06 ± 0.08	0.07 ± 0.07	0.05 ± 0.09	0.066
NLR	5.6 ± 3.8	6.6 ± 4.1	3.3 ± 1.7	0.001
PLR	190.0 ± 141.7	222.9 ± 152.8	110.9 ± 61.2	0.002
SII	1520.7 ± 1400.9	1840.0 ± 1544.7	754.4 ± 376.7	0.003
SIRI	4.2 ± 3.9	4.8 ± 4.5	2.7 ± 1.3	0.046
AISI	1158.2 ± 1461	1385.1 ± 1686.8	625.2 ± 325.2	0.051
ALBI	−2.7 ± 0.4	−2.6 ± 0.3	−2.6 ± 0.5	0.392
PALBI score	−2.4 ± 0.4	−2.4 ± 0.3	−2.5 ± 0.5	0.232

Abbreviations: IL-6, interleukine-6; CRP, C reactive protein; LMR. Lymphocyte-to-Monocyte Ratio; NLR. Neutrophil-to-Lymphocyte Ratio; PLR. Platelet-to-Lymphocyte Ratio; SII. Systemic Immune-Inflammation Index; SIRI. Systemic Inflammation Response Index; AISI. Aggregate Index of Systemic Inflammation; ALBI. Albumin-Bilirubin Score; PALBI score. Platelet-Albumin-Bilirubin Score.

**Table 6:** Immunophenotype of circulating T lymphocytes.

Variable	All Patients	BMI < 30 50 (71.4%)	BMI ≥ 30 20 (28.6%)	*p* Value
CD3+ T (number)	1413.2 ± 1849.1	862.22 ± 607.7	2515.3 ± 2877.1	0.025
CD3+ CD4+ T (number)	841.9 ± 1049.6	538.2 ± 442.8	1449 ± 1594.1	0.030
CD3+ CD8+T (number)	410.9 ± 1049.6	227.3 ± 214.6	778.0 ± 1003.8	0.032
CD3+ CD4+ CD25hi CD127-Treg (percentage)	0.6 ± 0.8	0.7 ± 0.9	0.5 ± 0.5	0.725
CD3+ CD4+ CD194+ CD196+ Th17 (percentage)	15.0 ± 16.6	12.3 ± 12.5	20.4 ± 22.7	0.268
Treg/Th17 ratio	0.09 ± 0.16	0.11 ± 0.18	0.05 ± 0.09	0.450

**Table 7:** Association between viral detection and obesity status (BMI < 30 vs. BMI > 30). Odds ratios (ORs), 95% confidence intervals (CIs), and *p*-values are reported.

Virus	BMI < 30		BMI ≥ 30		OR (95% CI)	*p*-Value
	Yes	No	Yes	No		
**Anelloviruses**						
TTV ^a^	36 (72.00%)	14 (28.00%)	9 (45.00%)	11 (55.00%)	0.32 (0.11–0.93)	0.04
**Polyomaviruses**						
BKV	16 (32.00%)	28 (68.00%)	4 (20.00%)	13 (65.00%)	0.54 (0.15–1.92)	0.34
JCV	21 (42.00%)	23 (46.00%)	10 (50.00%)	7 (35.00%)	1.56 (0.51–4.76)	0.45
**Adenoviruses**						
ADENO-11	37 (74.00%)	7 (14.00%)	12 (60.00%)	5 (25.00%)	0.45 (0.12–1.69)	0.30
ADENO-34	0 (0.00%)	44 (88.00%)	0 (0.00%)	17 (85.00%)	2.56 (0.10–100.00)	1.00
ADENO-35	0 (0.00%)	42 (84.00%)	2 (10.00%)	17 (85.00%)	12.50 (0.56–∞)	0.20
**Human Herpesviruses**						
HHV7	0 (0.00%)	44 (88.00%)	0 (0.00%)	17 (85.00%)	2.56 (0.10–100.00)	1.00
EBV	2 (4.00%)	42 (84.00%)	0 (0.00%)	17 (85.00%)	0.49 (0.02–11.11)	0.60
CMV	10 (20.00%)	34 (68.00%)	3 (15.00%)	14 (70.00%)	0.73 (0.17–3.03)	0.70
HSV-1	6 (12.00%)	38 (76.00%)	5 (25.00%)	12 (60.00%)	2.63 (0.68–10.00)	0.20
VZV	20 (40.00%)	24 (48.00%)	10 (50.00%)	7 (35.00%)	1.72 (0.56–5.26)	0.30
**Influenza virus**						
H1N1	0	0	0	0	----	----

^a^. for TTV a value of viremia < 4 log_10_/mL was considered as “No”. For the other viruses. we reported presence (Y) or absence (N). OR reflects the possibility of patients with BMI ≥ 30 of being positive for a certain virus. compared to BMI < 30 patients.

## Data Availability

The data that support the findings of this study are openly available in Zenodo at https://doi.org/10.5281/zenodo.18386515.

## Abbreviations

The following abbreviations are used in this manuscript:

6MWT	6 Minute walking Test
8-OHdG	8′-hydroxy-2′-deoxyguanosine
Ad11	Adenovirus 11
Ad34	Adenovirus 34
Ad35	Adenovirus 35
ADL	Activities of Daily Living
AISI	Aggregate Index of Systemic Inflammation
ALBI	Albumin-Bilirubin Score
ALT	alanine aminotransferase
AST	aspartate aminotransferase
BDI-II	Beck Depression Inventory-II
BKPyV	BK Polyoma Virus
BMI	Body Mass Index
CD	Cluster of Differentiation
CES-D	Center for Epidemiologic Studies Depression Scale
CIRS	Cumulative Illness Rating Scale
CMV	Cytomegalovirus
COPD	Chronic Obstructive Pulmonary Disease
CRP	C reactive protein
DMSO	Dimethylsulfoxide
EBV	Epstein–Barr Virus
ESR	Erythrosedimentation rate
FBS	Foetal Bovine Serum
FEV1/FVC%	Forced Expiratory Volume in 1 s/Forced Vital Capacity
γGT	gamma-glutamyl transferase
GOLD	Global initiative for chronic Obstructive Lung Diseases
HHV-6	Human Herpes Virus-6
HHV-7	Human Herpes Virus-7
HSV-1	Human Herpes Simples-1
IADL	Instrumental Activities of Daily Living
IL-6	interleukine-6
JCPyV	JC Polyoma Virus
MDA	malondialdehyde
MLR	Monocytes to Lymphocytes Ratio
MMSE	Mini-Mental State Examination
MRC	modified Medical Research Council (Dyspnoea Scale)
MRF-26	Maugeri Respiratory Failure 26 questions
NLR	Neutrophils to Lymphocytes Ratio
PALBI	Platelet-Albumin-Bilirubin Score
PBMC	Peripheral Blood Mononuclear Cells
PBS	Phosphate-Buffered Saline
PLR	Platelets to Lymphocytes Ratio
PLT	platelets
PR	Pulmonary Rehabilitation
QoL	Quality of Life
RBC	Red Blood Cells
SF-36	36-Item Short Form Health Survey—General Health domain;
SII	Systemic Immune Inflammation Index
SIRI	Systemic Inflammation Response Index
TTV	Torque Teno Virus
VZV	Varicella Zoster Virus
WBC	white blood cells
ZUNG	Zung Self-Rating Depression Scale

## References

[1] Ng M., Gakidou E., Lo J., Abate Y.H., Abbafati C., Abbas N., Abbasian M., Abd ElHafeez S., Abdel-Rahman W.M., Abd-Elsalam S. (2025). Global, Regional, and National Prevalence of Adult Overweight and Obesity, 1990–2021, with Forecasts to 2050: A Forecasting Study for the Global Burden of Disease Study 2021. Lancet.

[2] Edwards C.H., Aas E., Kinge J.M. (2019). Body Mass Index and Lifetime Healthcare Utilization. BMC Health Serv. Res..

[3] Nyberg S.T., Frank P., Ahmadi-Abhari S., Pentti J., Vahtera J., Ervasti J., Suominen S.B., Strandberg T.E., Sipilä P.N., Meri S. (2026). Adult Obesity and Risk of Severe Infections: A Multicohort Study with Global Burden Estimates. Lancet.

[4] Gao M., Piernas C., Astbury N.M., Hippisley-Cox J., O’Rahilly S., Aveyard P., Jebb S.A. (2021). Associations between Body-Mass Index and COVID-19 Severity in 6·9 Million People in England: A Prospective, Community-Based, Cohort Study. Lancet Diabetes Endocrinol..

[5] Muscogiuri G., Pugliese G., Laudisio D., Castellucci B., Barrea L., Savastano S., Colao A. (2021). The Impact of Obesity on Immune Response to Infection: Plausible Mechanisms and Outcomes. Obes. Rev..

[6] Fröhlich E. (2025). The Effects of Obesity on Lung Physiology, the Prevalence and Severity of Chronic Pulmonary Diseases, and Inhalation Treatment. Drug Des. Dev. Ther..

[7] Zerah F., Harf A., Perlemuter L., Lorino H., Lorino A.-M., Atlan G. (1993). Effects of Obesity on Respiratory Resistance. Chest.

[8] Jones R.L., Nzekwu M.-M.U. (2006). The Effects of Body Mass Index on Lung Volumes. Chest.

[9] Littleton S.W., Tulaimat A. (2017). The Effects of Obesity on Lung Volumes and Oxygenation. Respir. Med..

[10] McGinn E.A., Mandell E.W., Smith B.J., Duke J.W., Bush A., Abman S.H. (2023). Dysanapsis as a Determinant of Lung Function in Development and Disease. Am. J. Respir. Crit. Care Med..

[11] Green M., Mead J., Turner J.M. (1974). Variability of Maximum Expiratory Flow-Volume Curves. J. Appl. Physiol..

[12] Elliot J.G., Donovan G.M., Wang K.C.W., Green F.H.Y., James A.L., Noble P.B. (2019). Fatty Airways: Implications for Obstructive Disease. Eur. Respir. J..

[13] Wang C.J., Noble P.B., Elliot J.G., Choi Y.S., James A.L., Wang K.C.W. (2023). Distribution, Composition, and Activity of Airway-Associated Adipose Tissue in the Porcine Lung. Am. J. Physiol. Lung Cell. Mol. Physiol..

[14] Lestari I.P., Chozin I.N., Sartono T.R., Sasiarini L., Yudhanto H.S. (2024). Effect of a High-Calorie Diet on pro- to Anti-Inflammatory Macrophage Ratio through Fat Accumulation in Rat Lung Tissue. Med. J. Indones..

[15] Torday J.S., Powell F.L., Farmer C.G., Orgeig S., Nielsen H.C., Hall A.J. (2010). Leptin Integrates Vertebrate Evolution: From Oxygen to the Blood–Gas Barrier. Respir. Physiol. Neurobiol..

[16] Lv Y.-Q., Dhlamini Q., Chen C., Li X., Bellusci S., Zhang J.-S. (2021). FGF10 and Lipofibroblasts in Lung Homeostasis and Disease: Insights Gained From the Adipocytes. Front. Cell Dev. Biol..

[17] Martin M., Almeras N., Després J.-P., Coxson H., Washko G., Vivodtzev I., Wouters E., Rutten E., Williams M., Murchison J. (2017). Ectopic Fat Accumulation in Patients with COPD: An ECLIPSE Substudy. Int. J. Chronic Obstr. Pulm. Dis..

[18] Tanaka Y., Fujisawa T., Yazawa S., Ohta I., Takaku Y., Ito M., Inoue Y., Yasui H., Hozumi H., Karayama M. (2024). Obesity Impairs Ciliary Function and Mucociliary Clearance in the Murine Airway Epithelium. Am. J. Physiol. Lung Cell. Mol. Physiol..

[19] Hornung F., Rogal J., Loskill P., Löffler B., Deinhardt-Emmer S. (2021). The Inflammatory Profile of Obesity and the Role on Pulmonary Bacterial and Viral Infections. Int. J. Mol. Sci..

[20] Oppenheimer B.W., Berger K.I., Ali S., Segal L.N., Donnino R., Katz S., Parikh M., Goldring R.M. (2016). Pulmonary Vascular Congestion: A Mechanism for Distal Lung Unit Dysfunction in Obesity. PLoS ONE.

[21] Watanabe K., Suzukawa M., Arakawa S., Kobayashi K., Igarashi S., Tashimo H., Nagai H., Tohma S., Nagase T., Ohta K. (2019). Leptin Enhances Cytokine/Chemokine Production by Normal Lung Fibroblasts by Binding to Leptin Receptor. Allergol. Int..

[22] Ihrie M.D., McQuade V.L., Womble J.T., Hegde A., McCravy M.S., Lacuesta C.V.G., Tighe R.M., Que L.G., Walker J.K.L., Ingram J.L. (2022). Exogenous Leptin Enhances Markers of Airway Fibrosis in a Mouse Model of Chronic Allergic Airways Disease. Respir. Res..

[23] Bidan C.M., Veldsink A.C., Meurs H., Gosens R. (2015). Airway and Extracellular Matrix Mechanics in COPD. Front. Physiol..

[24] Peters U., Dixon A.E., Forno E. (2018). Obesity and Asthma. J. Allergy Clin. Immunol..

[25] Salome C.M., King G.G., Berend N. (2010). Physiology of Obesity and Effects on Lung Function. J. Appl. Physiol..

[26] Plopper C.G., Nishio S.J., Schelegle E.S. (2003). Tethering Tracheobronchial Airways within the Lungs. Am. J. Respir. Crit. Care Med..

[27] Srikanthan P., Seeman T.E., Karlamangla A.S. (2009). Waist-Hip-Ratio as a Predictor of All-Cause Mortality in High-Functioning Older Adults. Ann. Epidemiol..

[28] Masa J.F., Pépin J.-L., Borel J.-C., Mokhlesi B., Murphy P.B., Sánchez-Quiroga M.Á. (2019). Obesity Hypoventilation Syndrome. Eur. Respir. Rev..

[29] Vozoris N.T., Stanbrook M.B. (2011). Smoking Prevalence, Behaviours, and Cessation among Individuals with COPD or Asthma. Respir. Med..

[30] Fuller-Thomson E., Howden K.E.N., Fuller-Thomson L.R., Agbeyaka S. (2018). A Strong Graded Relationship between Level of Obesity and COPD: Findings from a National Population-Based Study of Lifelong Nonsmokers. J. Obes..

[31] Franssen F.M.E., O’Donnell D.E., Goossens G.H., Blaak E.E., Schols A.M.W.J. (2008). Obesity and the Lung: 5 · Obesity and COPD. Thorax.

[32] Zhang S., Lin Y., Fang Z., Wu X., Jiang J., Zeng Y., Liu J., Li J., Wang K., Song X. (2025). Association between Visceral Adiposity Index and Chronic Obstructive Pulmonary Disease: A Cross-Sectional Analysis. Sci. Prog..

[33] Jee S.H., Sull J.W., Park J., Lee S.-Y., Ohrr H., Guallar E., Samet J.M. (2006). Body-Mass Index and Mortality in Korean Men and Women. N. Engl. J. Med..

[34] Landbo C., Prescott E., Lange P., Vestbo J., Almdal T.P. (1999). Prognostic Value of Nutritional Status in Chronic Obstructive Pulmonary Disease. Am. J. Respir. Crit. Care Med..

[35] Purcell M., Ackland J., Staples K.J., Freeman A., Wilkinson T.M.A. (2025). The Respiratory Tract Virome: Unravelling the Role of Viral Dark Matter in Respiratory Health and Disease. Eur. Respir. Rev..

[36] Morgan O.W., Bramley A., Fowlkes A., Freedman D.S., Taylor T.H., Gargiullo P., Belay B., Jain S., Cox C., Kamimoto L. (2010). Morbid Obesity as a Risk Factor for Hospitalization and Death Due to 2009 Pandemic Influenza A(H1N1) Disease. PLoS ONE.

[37] Louie J.K., Acosta M., Samuel M.C., Schechter R., Vugia D.J., Harriman K., Matyas B.T., the California Pandemic (H1N1) Working Group (2011). A Novel Risk Factor for a Novel Virus: Obesity and 2009 Pandemic Influenza A (H1N1). Clin. Infect. Dis..

[38] Van Kerkhove M.D., Mounts A.W., Mall S., Vandemaele K.A.H., Chamberland M., Dos Santos T., Fitzner J., Widdowson M.-A., Michalove J., Bresee J. (2011). Epidemiologic and Virologic Assessment of the 2009 Influenza A (H1N1) Pandemic on Selected Temperate Countries in the Southern Hemisphere: Argentina, Australia, Chile, New Zealand and South Africa: Southern Hemisphere H1N1pdm. Influenza Other Respir. Viruses.

[39] Coleman L.A., Waring S.C., Irving S.A., Vandermause M., Shay D.K., Belongia E.A. (2013). Evaluation of Obesity as an Independent Risk Factor for Medically Attended Laboratory-confirmed Influenza. Influenza Resp. Viruses.

[40] Cilloniz C., Luna C.M., Hurtado J.C., Marcos M.Á., Torres A. (2022). Respiratory Viruses: Their Importance and Lessons Learned from COVID-19. Eur. Respir. Rev..

[41] Focosi D., Antonelli G., Pistello M., Maggi F. (2016). Torquetenovirus: The Human Virome from Bench to Bedside. Clin. Microbiol. Infect..

[42] Maggi F., Bendinelli M. (2010). Human Anelloviruses and the Central Nervous System. Rev. Med. Virol..

[43] Görzer I., Haloschan M., Jaksch P., Klepetko W., Puchhammer-Stöckl E. (2014). Plasma DNA Levels of Torque Teno Virus and Immunosuppression after Lung Transplantation. J. Heart Lung Transplant..

[44] Albert E., Solano C., Giménez E., Focosi D., Pérez A., Macera L., Piñana J.L., Boluda J.C.H., Maggi F., Navarro D. (2018). The Kinetics of Torque Teno Virus Plasma DNA Load Shortly after Engraftment Predicts the Risk of High-Level CMV DNAemia in Allogeneic Hematopoietic Stem Cell Transplant Recipients. Bone Marrow Transpl..

[45] Fernández-Ruiz M., Albert E., Giménez E., Ruiz-Merlo T., Parra P., López-Medrano F., San Juan R., Polanco N., Andrés A., Navarro D. (2019). Monitoring of Alphatorquevirus DNA Levels for the Prediction of Immunosuppression-Related Complications after Kidney Transplantation. Am. J. Transplant..

[46] Vitiello L., Proietti S., Limongi D., Prezioso C., Checconi P., Fortugno P., Quaranta M., Maggi F., Antonelli G., Bonassi S. (2026). TTV Virome Marks Immune Exhaustion, α7nAChR Alteration, and Mortality in Elderly Patients with Severe COPD. GeroScience.

[47] (2015). Quality Management Systems—Requirements.

[48] (2016). Medical Devices—Quality Management Systems—Requirements for Regulatory Purposes.

[49] Ilari S., Vitiello L., Russo P., Proietti S., Milić M., Muscoli C., Cardaci V., Tomino C., Bonassi G., Bonassi S. (2021). Daily Vegetables Intake and Response to COPD Rehabilitation. The Role of Oxidative Stress, Inflammation and DNA Damage. Nutrients.

[50] Bonassi S., Ceppi M., Møller P., Azqueta A., Milić M., Neri M., Brunborg G., Godschalk R., Koppen G., Langie S.A.S. (2021). DNA Damage in Circulating Leukocytes Measured with the Comet Assay May Predict the Risk of Death. Sci. Rep..

[51] Milić M., Ceppi M., Bruzzone M., Azqueta A., Brunborg G., Godschalk R., Koppen G., Langie S., Møller P., Teixeira J.P. (2021). The hCOMET Project: International Database Comparison of Results with the Comet Assay in Human Biomonitoring. Baseline Frequency of DNA Damage and Effect of Main Confounders. Mutat. Res. Rev. Mutat. Res..

[52] Brucklacher-Waldert V., Steinbach K., Lioznov M., Kolster M., Hölscher C., Tolosa E. (2009). Phenotypical Characterization of Human Th17 Cells Unambiguously Identified by Surface IL-17A Expression. J. Immunol..

[53] Coursey T.G., Gandhi N.B., Volpe E.A., Pflugfelder S.C., De Paiva C.S. (2013). Chemokine Receptors CCR6 and CXCR3 Are Necessary for CD4+ T Cell Mediated Ocular Surface Disease in Experimental Dry Eye Disease. PLoS ONE.

[54] Ali S., Fevery J., Peerlinck K., Verslype C., Schelstraete R., Gyselinck F., Emonds M., Vermylen J., Hiem Yap S. (2002). TTV Infection and Its Relation to Serum Transaminases in Apparently Healthy Blood Donors and in Patients with Clotting Disorders Who Have Been Investigated Previously for Hepatitis C Virus and GBV-C/HGV Infection in Belgium. J. Med. Virol..

[55] Russo P., Milani F., Limongi D., Prezioso C., Novazzi F., Ferrante F.D., Maggi F., Antonelli G., Bonassi S. (2025). The Effect of Torque Teno Virus (TTV) Infection on Clinical Outcomes, Genomic Integrity, and Mortality in COPD Patients. Mech. Ageing Dev..

[56] Focosi D., Spezia P.G., Macera L., Salvadori S., Navarro D., Lanza M., Antonelli G., Pistello M., Maggi F. (2020). Assessment of Prevalence and Load of Torquetenovirus Viraemia in a Large Cohort of Healthy Blood Donors. Clin. Microbiol. Infect..

[57] Giacconi R., Maggi F., Macera L., Pistello M., Provinciali M., Giannecchini S., Martelli F., Spezia P.G., Mariani E., Galeazzi R. (2018). Torquetenovirus (TTV) Load Is Associated with Mortality in Italian Elderly Subjects. Exp. Gerontol..

[58] Russo P., Milani F., De Iure A., Proietti S., Limongi D., Prezioso C., Checconi P., Zagà V., Novazzi F., Maggi F. (2024). Effect of Cigarette Smoking on Clinical and Molecular Endpoints in COPD Patients. Int. J. Mol. Sci..

[59] Yao S., Zeng L., Wang F., Chen K. (2023). Obesity Paradox in Lung Diseases: What Explains It?. Obes. Facts.

[60] Chittal P., Babu A.S., Lavie C.J. (2015). Obesity Paradox: Does Fat Alter Outcomes in Chronic Obstructive Pulmonary Disease?. COPD J. Chronic Obstr. Pulm. Dis..

[61] Cao C., Wang R., Wang J., Bunjhoo H., Xu Y., Xiong W. (2012). Body Mass Index and Mortality in Chronic Obstructive Pulmonary Disease: A Meta-Analysis. PLoS ONE.

[62] Gorecka D., Gorzelak K., Sliwinski P., Tobiasz M., Zielinski J. (1997). Effect of Long-Term Oxygen Therapy on Survival in Patients with Chronic Obstructive Pulmonary Disease with Moderate Hypoxaemia. Thorax.

[63] Blum A., Simsolo C., Sirchan R., Haiek S. (2011). “Obesity Paradox” in Chronic Obstructive Pulmonary Disease. Isr. Med. Assoc. J..

[64] Brigham E.P., Anderson J.A., Brook R.D., Calverley P.M.A., Celli B.R., Cowans N.J., Crim C., Diserens J.E., Martinez F.J., McCormack M.C. (2021). Challenging the Obesity Paradox: Extreme Obesity and COPD Mortality in the SUMMIT Trial. ERJ Open Res..

[65] Sun Y., Milne S., Jaw J.E., Yang C.X., Xu F., Li X., Obeidat M., Sin D.D. (2019). BMI Is Associated with FEV1 Decline in Chronic Obstructive Pulmonary Disease: A Meta-Analysis of Clinical Trials. Respir. Res..

[66] Wagner P.D. (2008). Possible Mechanisms Underlying the Development of Cachexia in COPD. Eur. Respir. J..

[67] Yu X.-Y., Song P., Zou M.-H. (2018). Obesity Paradox and Smoking Gun: A Mystery of Statistical Confounding?. Circ. Res..

[68] Carbone S., Lavie C.J., Arena R. (2017). Obesity and Heart Failure: Focus on the Obesity Paradox. Mayo Clin. Proc..

[69] Marquis K., Debigaré R., Lacasse Y., LeBlanc P., Jobin J., Carrier G., Maltais F. (2002). Midthigh Muscle Cross-Sectional Area Is a Better Predictor of Mortality than Body Mass Index in Patients with Chronic Obstructive Pulmonary Disease. Am. J. Respir. Crit. Care Med..

[70] Wouters E.F.M. (2017). Obesity and Metabolic Abnormalities in Chronic Obstructive Pulmonary Disease. Ann. Am. Thorac. Soc..

[71] Sweatt K., Garvey W.T., Martins C. (2024). Correction: Strengths and Limitations of BMI in the Diagnosis of Obesity: What Is the Path Forward?. Curr. Obes. Rep..

[72] Stocker R., Yamamoto Y., McDonagh A.F., Glazer A.N., Ames B.N. (1987). Bilirubin Is an Antioxidant of Possible Physiological Importance. Science.

[73] MacDonald D.M., Kunisaki K.M., Wilt T.J., Baldomero A.K. (2021). Serum Bilirubin and Chronic Obstructive Pulmonary Disease (COPD): A Systematic Review. BMC Pulm. Med..

[74] Khudhur Z.O., Smail S.W., Awla H.K., Ahmed G.B., Khdhir Y.O., Amin K., Janson C. (2025). The Effects of Heavy Smoking on Oxidative Stress, Inflammatory Biomarkers, Vascular Dysfunction, and Hematological Indices. Sci. Rep..

[75] Rabrenović V., Petrović M., Rabrenović M., Pilčević D., Rančić N. (2024). The Significance of Biomarkers of Inflammation in Predicting the Activity of Lupus Nephritis. J. Med. Biochem..

[76] Hu B., Yang X.-R., Xu Y., Sun Y.-F., Sun C., Guo W., Zhang X., Wang W.-M., Qiu S.-J., Zhou J. (2014). Systemic Immune-Inflammation Index Predicts Prognosis of Patients after Curative Resection for Hepatocellular Carcinoma. Clin. Cancer Res..

[77] Zinellu A., Collu C., Nasser M., Paliogiannis P., Mellino S., Zinellu E., Traclet J., Ahmad K., Mangoni A.A., Carru C. (2021). The Aggregate Index of Systemic Inflammation (AISI): A Novel Prognostic Biomarker in Idiopathic Pulmonary Fibrosis. J. Clin. Med..

[78] Jia S., Chen Q., Huang W., Wang P., Zeng Y. (2025). Relationship between Systemic Immune Response Index (SIRI) and COPD: A Cross-Sectional Study Based on NHANES 2007–2012. Sci. Rep..

[79] Song N., Elbahnasawy M.A., Weng N.-P. (2025). General and Individualized Changes in T Cell Immunity during Aging. J. Immunol..

[80] Trim W.V., Lynch L. (2022). Immune and Non-Immune Functions of Adipose Tissue Leukocytes. Nat. Rev. Immunol..

[81] Tilg H., Ianiro G., Gasbarrini A., Adolph T.E. (2025). Adipokines: Masterminds of Metabolic Inflammation. Nat. Rev. Immunol..

[82] Babakhani K., Kucinskas A.L., Ye X., Giles E.D., Sun Y. (2025). Aging Immunity: Unraveling the Complex Nexus of Diet, Gut Microbiome, and Immune Function. Immunometabolism.

[83] Curtis M., Swan L., Fox R., Warters A., O’Sullivan M. (2023). Associations between Body Mass Index and Probable Sarcopenia in Community-Dwelling Older Adults. Nutrients.

[84] Badillo-Pazmay G.V., Fortunato C., Cianfruglia L., Novazzi F., Spezia P.G., Rosa L., Limongi D., Prezioso C., D’Argenio V., Scudiero O. (2026). The Gut and Circulating Virome: Emerging Players in Aging and Longevity. Front. Aging.

[85] Lv P., Zhao X., Zhang H., Lu Q., Liang X. (2026). Nomogram Integrating Inflammatory Biomarkers Predicts Chronic Obstructive Pulmonary Disease Exacerbation Post-Rehabilitation. BMC Pulm. Med..

[86] Lazio Regional Health Service (SSR) (2016). Riorganizzazione Dei Percorsi Riabilitativi in Ambito Ospedaliero e Territoriale. Lazio Regional Health Service Guidelines.

